# Implementation of Antibiotic Discovery by Student Crowdsourcing in the Valencian Community Through a Service Learning Strategy

**DOI:** 10.3389/fmicb.2020.564030

**Published:** 2020-11-16

**Authors:** Sergi Maicas, Belén Fouz, Àngela Figàs-Segura, Jesús Zueco, Hortensia Rico, Alfonso Navarro, Ester Carbó, Jaume Segura-García, Elena G. Biosca

**Affiliations:** ^1^Departamento de Microbiología y Ecología, Facultad de Ciencias Biológicas, Universitat de València, Valencia, Spain; ^2^Estructura de Investigación Interdisciplinar en Biotecnología y Biomedicina, Universitat de València, Valencia, Spain; ^3^Departamento de Microbiología y Ecología, Facultad de Farmacia, Universitat de València, Valencia, Spain; ^4^Departamento de Microbiología y Ecología, Facultad de Medicina y Odontología, Universitat de València, Valencia, Spain; ^5^Departamento de Biología Vegetal, Área de Edafología y Química Agrícola, Facultad de Farmacia, Universitat de València, Valencia, Spain; ^6^Departamento de Calidad Ambiental y Suelos, Centro de Investigaciones sobre Desertificación-CIDE, Universitat de València-Centro Superior de Investigaciones Científicas-Generalitat Valenciana (UVEG-CSIC-GV) Moncada, Valencia, Spain; ^7^Departamento de Informática, Escuela Técnica Superior de Ingeniería, Universitat de València, Valencia, Spain

**Keywords:** antibiotic discovery, student crowdsourcing, antimicrobial resistance, service learning, soil bacteria, geolocation, Natura project, gender equality

## Abstract

Antibiotic misuse is a public health problem due to the appearance of resistant strains in almost all human pathogens, making infectious diseases more difficult to treat. The search for solutions requires the development of new antimicrobials as well as novel strategies, including increasing social awareness of the problem. The Small World Initiative (SWI) and the Tiny Earth (TE) network are citizen science programs pursuing the discovery of new antibiotics from soil samples and the promotion of scientific culture. Both programs aim to bring scientific culture and microbiological research closer to pre-university students through a crowdsourcing strategy and a Service Learning (SL) educational approach, with a 2-fold objective: to encourage students to pursue careers in science and to involve them in the discovery of soil microorganisms producing new antimicrobials. SWI and TE projects were put into practice in Spain under the common name *MicroMundo*. *MicroMundo*@Valencia was implemented at the Universitat de València (UV) during the academic years 2017–2018 and 2018–2019. It trained 140 university students to disseminate this initiative into 23 high/secondary schools, and one primary school, involving about 900 people (teachers and students) as researchers. A total of 7,002 bacterial isolates were obtained from 366 soil samples and tested for antibiosis at UV and high/secondary school centers. About 1 or 7% of them produced inhibition halos for the *Escherichia coli* or *Bacillus cereus* target strains, respectively. Geolocation of sampling sites by an application developed *ad hoc* and Kriging analysis also allowed detection of soil foci of antibiotic-producing bacteria. Evaluation of the project by university, high/secondary, and primary school students revealed their strong positive perception and their increased interest in science, as a consequence of acquiring new scientific and pedagogical concepts and skills that they were able to pass on to other classmates, younger students, or relatives. To further expand the dissemination of the project in the Valencian Community, diverse extramural activities deemed to include a gender perspective and aimed at different age groups, were also carried out, obtaining very satisfactory results, increasing sensitivity and awareness to the global antibiotic crisis.

## Introduction

Arguably, antibiotics are one of the greatest discoveries of modern medicine and, during the first decade, carried away by the success, health professionals were not aware of the fragility of that medical breakthrough. However, the phenomenon of antibiotic resistance was known from the very beginning: Penicillin was introduced into clinical use in 1941, but its inactivation by an *Escherichia coli* strain with penicillin-hydrolyzing enzymatic activity had already been reported 1 year before (Abraham and Chain, 1940) and by 1942, penicillinase-producing strains of *Staphylococcus aureus* were found in hospitalized patients (Rammelkamp and Maxon, 1942). It could then be said that antibiotics, in a way, carried the potential for their own downfall. To make matters worse, the misuse (for either unnecessary, inappropriate, or both), the overuse of antibiotics, and the improper level of microbial exposure (mode and timing of administration, dose, dosing interval, etc.) when treating patients over the last almost 80 years, have taken us to the brink of losing the war against bacterial pathogens.

The outcome of all these factors is the situation we face today, in which the emergence of antibiotic-resistant strains across almost all human pathogens is wreaking havoc (Burnham et al., 2019). Undeniably, the solution not only requires the development of new antimicrobial drugs acting through new mechanisms and aiming to new targets (Mulani et al., 2019; Wang et al., 2020) but it also demands additional new strategies able to pave the way to move forward, like the establishment of adequate antibiotic stewardship policies within the medical community as well as raising social awareness of the magnitude of the problem we have to tackle.

This is where initiatives such as the Small World Initiative (SWI), a citizen science project pursuing to discover new antibiotics and promote scientific culture, that originated in 2012 at Yale University (United States; Hernandez et al., 2015), or the Tiny Earth (TE) network, an educational program headed by the UW-Madison’s Wisconsin Institute for Discovery (United States), initiated in 2018, that strives to forge a network of instructors and students focused on student-sourcing antibiotic discovery from soil, may become crucial when it comes to raising the much needed community awareness. Both proposals address the pandemic spread of antibiotic resistance by targeting at individual-level two of its structural roots, namely, the current shortage in the development of new therapeutic antimicrobial drugs and the lack of awareness and understanding of the problem within the general population.

Both programs aim to bring scientific culture and biomedical research closer to pre-university education levels through a crowdsourcing strategy, aimed at discovering novel microorganisms producing new antibiotics from soil samples. Emulating the discovery of penicillin by Alexander Fleming, but in an organized and participatory way, the programs seek to involve a large group of volunteers or a community, through an open call, with a 2-fold objective. On the one hand, they aim to encourage students to pursue careers in science, as well as to entice them to become the scientists of tomorrow (Caruso et al., 2016; Davis et al., 2017). In addition, by involving citizenry in the discovery process, they greatly increase the chances of finding new bioactive molecules, which might prove to be useful therapeutics. Both initiatives are currently underway at different educational levels, in at least 15 countries and involving tens of thousands of students.

To engage students in the SWI and Tiny Earth research experiences in microbiology, the innovative educational strategy Service Learning (SL; Esson and Stevens-Truss, 2005; Webb, 2017) was used. The SL is an educational proposal that develops both learning procedures and community service in a single project since, by connecting motivation with experimentation, the training of the students is enhanced, and they in turn are involved in the real needs of their close environment with the aim of improving it. This learning strategy brings many pedagogical and social benefits to the students involved, improving their self-learning, academic and civic engagement, social skills, and moral reasoning (Esson and Stevens-Truss, 2005; Celio et al., 2011; Lies et al., 2012; Webb, 2017).

In July 2017, the SWI@Spain network was created at the *Universidad Complutense de Madrid* (UCM), sponsored by the group of Teaching and Dissemination of Microbiology (D+DM) of the Spanish Society for Microbiology (SEM; Valderrama et al., 2018), in which SWI@Valencia from the *Universitat de València* (UV) participated as a foundational member.^1^ Currently, 31 groups are working in different universities from the Iberian Peninsula (Spain and Portugal), under the common name *MicroMundo*, joining the efforts of nearly 100 instructors and an even higher number of secondary education schools.

The *MicroMundo*@Valencia initiative was implemented at the UV in the academic course 2017–2018, similarly to SWI@Spain/*MicroMundo* at the UCM (Valderrama et al., 2018). Likewise, the main objectives were: (i) to generate sensitivity toward antibiotic resistance and the need to spread awareness of the problem to the society at different educational levels, (ii) to awaken the interest in science through experiential learning and student discovery of bacteria that produce new antibiotics, and (iii) to encourage scientific training and learning in science by mutual cooperation among students of different education levels through a community service.

In the 2018–2019 course, *MicroMundo*@Valencia also traveled to primary schools, within the framework of the “*Natura* projects” program of the UV. This innovative program tries to unite the work done by different natural science projects and to improve the teaching quality using SL as educational strategy by training students of different educational levels scientifically through adapted sessions. The interest of the project resides in the fact that teaching is given by university students to high school students and subsequently by the latter to primary school students. It is a good educational tool since university students reinforce their knowledge by being responsible for transmitting it to high/secondary school students, while the latter learn new concepts that can also be transmitted to primary school students. Thus, the project promotes the connection of university students with those of high/secondary and primary schools which offers a unique opportunity for social and intellectual interactions between them hardly achievable in other ways (Abrahamsen, 2004). Moreover, the implementation of *Natura* projects in the different educational levels, implies the extension and complementation of the curricular content within the subjects of biology and geology for the high/secondary school level, and of natural sciences in the case of primary education.

Finally, to further extend the dissemination of the *MicroMundo*@Valencia initiative into the society, different extramural activities were carried out including participation in scientific fairs, gender equality, and infographic activities for high/secondary school students, scientific meetings, as well as in diverse community-wide actions to achieve significant media coverage. In this paper, we report the results of our participation as the *MicroMundo* group in Valencia’s Community, as well as in additional outreach activities outside the UV.

## Materials and Methods

### Implementation of *MicroMundo*@Valencia at the UV

Professors from the Departments of Microbiology and Ecology, Soil Science, and Computer Science of the UV, as well as other members of the Department of Microbiology and Ecology were involved in the project implementation. Microbiology professors (*MicroMundo* Partner Instructors, MMPIs) carried out the recruitment of university undergraduate students of microbiology of the different degrees and masters of the UV and taught them a training course to become monitors (*MicroMundo* Teaching Assistants, MMTAs; see below). MMPIs were also in charge of contacting, recruiting, and selecting the participating partners (public high/secondary education schools), as well as in obtaining funds from different UV sources. Science secondary teachers from public schools involved in this initiative also participated applying for funds to the local government. Additionally, in the context of the “*Natura* projects” program at UV, some MMPIs, one MMTA, and a group of high school students (see below) participated in a pilot project to adapt and transfer *MicroMundo*@Valencia to primary schools.

**Figure 1:**
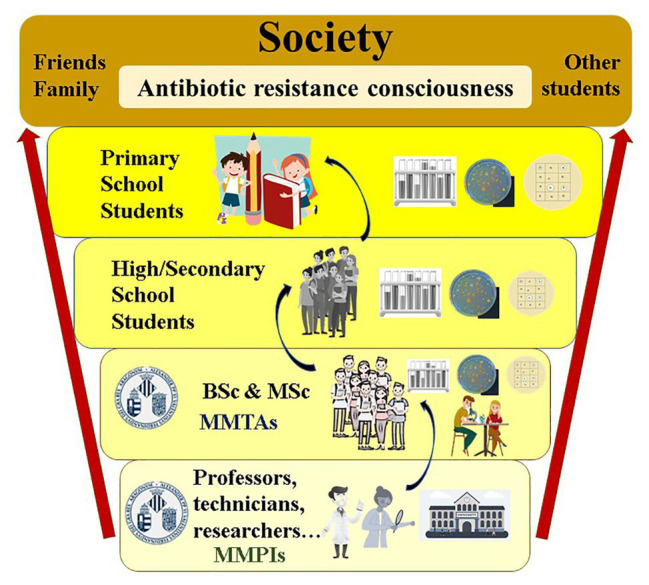
Scheme of progressive implementation of *MicroMundo*@Valencia at different educational levels, from university to primary school, in the Valencian Community.

A scheme of the progressive implementation of *MicroMundo*@Valencia at different educational levels in the Valencian Community is shown in Figure 1.

### Biosafe Practices

Since activities of this project involve working with live microorganisms, biosafety practices for teaching laboratories (Emmert, 2013) were adopted and taught to the students to safely work both, in Microbiology laboratories at UV and in science laboratories at schools, as well as to prevent the potential spread of the microorganisms. All students had disposable lab coats and gloves, and laboratory benches or classroom tables were disinfected both before and after each practical session. Further, the waste material derived from each session was collected and autoclaved at the UV facilities. Prior to science practices, all school students were authorized by their respective centers and parents or tutors.

### Training Courses for Monitors

Initially, a call was made to the students of different science degrees (Biochemistry and Biomedical Science, Biology, Biotechnology, Environmental Sciences, Food Science and Technology, and Pharmacy) and Masters (Molecular, Cellular and Genetics Biology, Secondary Education Teaching, and Food Quality and Safety) enrolled at the UV, as well as in the Master in Social Communication of Scientific Research at the *Universidad Internacional de Valencia*. The basis of the project was explained in a presentation talk, which was updated and exposed annually at the beginning of each course to attract new undergraduate students to the *MicroMundo*@Valencia team. Due to the large number of students who applied to participate in the project, a selection was made in order to carry out their training, in a coordinated manner, with the Microbiology MMPIs in three laboratories of the Faculty of Biological Sciences at UV, each academic year. MMTAS were trained through five theoretical-practical sessions in laboratories with the support of a manual adapted from Hernandez et al. (2015) and Valderrama et al. (2018). Briefly, these practical sessions included: (1) explanations on bacterial resistance, soil sampling, and use of a new geolocation application; (2) diluting and plating soil samples for isolation of microorganisms; (3) observation of soil microbial diversity, plate counts, and selection of colonies; (4) testing soil microorganisms for antibiosis activities; and (5) antibiosis observation, results interpretation, and surveys. The five sessions were similar to those held later in the schools, adapting the contents (see below). Both the educational and laboratory materials for the science practices in secondary schools were prepared by the MMTAs under the supervision of their corresponding MMPIs.

### Science Practices in High/Secondary Schools

Basically, the project consisted of five sessions lasting around 2 h separated by at least 2 days to allow the growth of soil microorganisms at room temperature. The sessions took place in the science laboratories of each one of the high/secondary schools participating in the project. Culture media for bacteria, sterile containers, and other materials were provided by the *MicroMundo*@Valencia teams. Students were also provided with an edited booklet with instructions. The content of each session is summarized below.

#### Session 1: Explanations on Bacterial Resistance, Soil Sampling, and Use of a New Geolocation Application

This first session started with the presentation and moderation of a debate on the problem of bacterial resistance to antibiotics and its socio-economic impact. For this, the different *MicroMundo*@Valencia teams used the same presentation in all high/secondary schools. This presentation also included a step by step description of the methodology. Each working group was made up by one MMPI and three to five MMTAs. Sampling material and instructions on how to choose a good sampling place were also provided at this session to groups of two or three students, which was normally programmed on a Friday to allow students to take a trip over the weekend to different places of Valencian Community to get soil samples. A mobile phone application specifically developed for this project downloaded the coordinates of the sampling site into a database. Further data related to encompassing features of the environment were provided by students through a fill-in form. Sterile containers were provided for both the microbiology and edaphology samples. Instructions were also provided on the important matter of the identification of the samples, using a code identifying the high/secondary school and the university team responsible for the sample, as well as for storage at room temperature.

#### Session 2: Diluting and Plating Soil Samples for Isolation of Microorganisms

Once edaphology samples were collected, work was started on the isolation of soil microorganisms from the microbiology samples. To this end, 9 ml of sterile water was added to 1 g of soil and serial 10-fold dilutions up to 10^−5^ were performed. One hundred microliter of the corresponding dilutions was spread on the surface of trypticase soy agar (Insulab, Valencia, Spain) diluted 1/10 (TSA 1/10, with agar up to 1.5%) plates using a Digralsky spreader and the plates were left to incubate at room temperature in a dark place. The addition of cycloheximide to TSA was ruled out due to the toxicity of this fungicide. In all this work, the secondary students were supervised by the MMTAs.

#### Session 3: Observation of Soil Microbial Diversity, Plate Counts, and Selection of Colonies

After 3 days of incubation, plates were inspected for having either well isolated colonies or between 20 and 200 colonies for plate counts. Individual colonies were transferred to fresh full-strength TSA plates using sterile toothpicks. Up to 20 individual colonies were transferred to a single plate placed over a grid template enabling easy colony coding and identification.

#### Session 4: Testing Soil Microorganisms for Antibiosis Activities

TSA plates were inoculated to saturation with a standard Gram positive bacterium, *Bacillus cereus* CECT 101, or a standard Gram negative bacterium, *E. coli* CECT 495, and left at 4°C overnight in the UV laboratory to prevent growth, before taking them back to the secondary schools. Students transferred the colonies isolated in the previous session onto the growing lawns of *E. coli* or *B. cereus* under the supervision of the MMTAs. For this, sterile toothpicks were used and the plates were placed on top of the template used in the previous session so that the distribution of the soil microorganisms on these plates matched the original distribution of their first isolation.

#### Session 5: Antibiosis Observation, Results Interpretation, and Surveys

After 2 days of incubation at room temperature, the plates were observed against a strong backlight in search of growth-inhibition halos identifying antibiosis phenomena. An interpretation of the results was made with the help of the MMTAs. Comparisons on the different effect of the same soil microorganism on Gram positive and Gram negative bacteria were made on the context of the spectrum of the possible antibiotics produced. The most promising soil microorganisms were transferred to fresh TSA plates to obtain pure culture for further characterization and study. Evaluation questionnaires were distributed to secondary school teachers and students and to MMTAs, and feedback was given to school teachers to prepare posters and videos for the closing day of the project (see below). Finally, all school students and teachers received a diploma to recognize and acknowledge their contribution.

### Science Practices in Primary Schools

Although the *MicroMundo*@Valencia project has traditionally focused on high/secondary education level, in the academic course 2018–2019, it was implemented in students of a local primary school through a *Natura* project at UV in order to spread awareness of the problem of antibiotic resistance from high/secondary school to elementary level. The thematic blocks related to the project for high/secondary education within the subjects of biology and geology were “Cell organization,” “Biodiversity,” and “Scientific methodology.” In the case of primary education, the blocks within natural sciences were “Human beings and health” and “Living beings.” Within each block, students could acquire and practice key competencies including, among others, language communication abilities, digital capacities, mathematical and basic skills in science and technology, as well as social and civic aptitudes.

Two classes of sixth grade of primary education (10–11 years old) of the school CEIP San Juan de Ribera (Burjassot, Valencia) participated in the project together with a group of high school students from the Vicent Andrés Estellés high school (Burjassot, Valencia). The high school students were trained by a MMTA, under the supervision of three MMPIs, to act as monitors for primary students through adapted sessions. For this purpose, four sessions were planned, the first two focused on the training of the high school students at their school, and the last two on the practical classes for the elementary students at their school. The practical classes were more approachable than those at the high school and adapted to this level (10–11 years old). The sessions are briefly explained below, summarizing their contents.

#### Session 1: Explanation About the Project

During this first session at the high school, *Natura* project was explained to high school student volunteers and recommendations about their role as instructors were made so that they could adapt the project contents to primary students level.

#### Session 2: High School Students’ Exposition and Evaluation

In this case, the powerpoint presentations and questions related to the project prepared in advance by the high school students were revised to check their suitability for the elementary students. These questions were prepared to assess the degree of understanding of these younger students about the new concepts and knowledge explained.

#### Session 3: Practical Class 1. Introduction to the Project. Culturing Soil Bacteria

This session took place at the primary school with groups of four to five students. First of all, the high school students explained to the younger ones the main important concepts to follow the rest of the activities, as well as biosafe measures to work with microorganisms. After that, students proceeded with the practical part with all the material being supplied by *Natura* project and participating MMPIs. Dilutions of soil samples were provided as well. Primary school students used a sterile swab that was introduced in one of the dilutions tubes and thereafter spread on the surface of one TSA plate, in the same way as it was done by MMTAs and high school students in the *MicroMundo*@Valencia project.

#### Session 4: Practical Class 2. Microbial Diversity and Antibiosis Observation

After 1 week of incubation, primary school students were able to see the bacterial diversity coming from the soil samples used. They observed diverse bacterial colonies on TSA plates, and were able to see natural antibiosis phenomena that some of them produced against other microorganisms present in the same plate. Moreover, and to complement the activities and the information given to the students, antibiosis assays against one strain of *B. cereus* were prepared at UV using bacterial colonies of the same soil samples. Plates with growth inhibition halos were shown to the elementary students in order they saw the microbial diversity in a soil sample, the antibiosis phenomenon among some of the soil bacteria, and the antibiosis against a specific bacterial strain. Thereafter, some questions were made to the primary students to favor their knowledge acquisition related to the project. Finally, high and primary school students and teachers received a diploma as an acknowledgment for their participation.

### Evaluation of MMTAs/Students and Science Practices

At the end of the practical sessions at the UV and the schools, anonymous surveys were filled by MMTAs and high/secondary and primary school students and teachers, adapting the questionnaire according to the involvement and educational level. Surveys presented different blocks and allowed responses based on a 5-value Likert scale, with 1 being the most negative and 5 the most positive (Likert, 1932). This scale was used to try to measure the attitudes, opinions, and perceptions of the different participating students regarding the problem of antibiotic resistance, how some of them could help to explain the problem to younger students and teach them practices on discovery of soil bacteria potentially producing antibiotics, and whether the accomplishment of these practices can improve their knowledge and awareness of a real problem, as well as increase their interest in science. In addition, an open block was included to provide opinions and make proposals for the improvement of project activities. Due to the anonymous nature of the surveys, the results could not be separated according to the gender of the students.

*MicroMundo* Teaching Assistants surveys consisted of four closed blocks to assess their opinion on the scientific interest of the project, stress the concept of antibiotic resistance, and expose their experience as teachers for younger students and on their collaboration in the project. The MMTAs survey was based on that of Valderrama et al. (2018) which, in turn, was based on the United States SWI Undergraduate Research Student Self-Assessment document (URSSA, SWI, United States). Surveys for high/secondary school students were simpler, with three closed blocks of questions on their scientific interest, their previous knowledge about antibiotic resistance, and their participation in the project. The secondary school teacher surveys consisted of two closed blocks on the interest of the project for their students and their personal opinion. Primary school student surveys were even shorter, with only six questions distributed in three blocks about their learning experience, their interest in the project, and their opinion about the high school monitors.

### Computer Science

An Android application (APP) was designed and developed to geolocate the collection sites of the soil samples, filling a form to characterize the site, and labeling each sample. This APP is available at the link https://www.uv.es/swi/swi.apk. Once downloaded and installed on the phone or tablet, the APP sends the information gathered with a form asking for the environmental conditions of the sample collection and it is sent using POST protocol to a spreadsheet in a Google server that gathers this information to be processed later. A guide was written to facilitate the use of the application as well.^2^

Also, the geolocation of the samples was used to draw a map of sampling sites, in order to be able to show all the information available from the form. The aim was that the processing of the samples provided information on the microorganisms able to inhibit the growth of control bacteria (*E. coli* and *B. cereus*). With this information, different maps were developed using spatial statistics techniques, such as Kriging technique (Wackernagel, 2003), in order to show the spatial statistical presence of these soil bacteria.

### Soil Science

Since it was important to know the characteristics of the soil and its environment in order to look for possible relationships with the production of antibiotics by soil microorganisms, a group of MMTAs participated in the soil session “Easily identifiable soil characteristics and their influence on its microbial inhabitants.” The goal was to learn the importance of the soil as a resource and identify some characteristics that may influence the type of soil microorganisms. The students worked in the Department’s Edaphology laboratory with the soil sample they had taken to do the *MicroMundo*@Valencia practice in the Microbiology laboratory. The size and number of the species diversity of soil microorganisms depend on various factors, mainly on the physical and chemical soil characteristics such as texture, humidity, pH, and organic matter content (Voroney, 2007; Mansour et al., 2015). These soil properties were determined according to the official laboratory methods of the Ministry of Agriculture, Fisheries, and Food (MAPA, 1994).

### Closing Day

All school students involved in the *MicroMundo*@Valencia project were invited to prepare posters, videos, or presentations about their results to show them in the closing day organized by the MMPIs at UV. The event started with the presentation of different political and academic authorities contributing to the project. Afterward, different activities were run, photocall, poster, and video competitions for the high/secondary school students as well as a Kahoot competition^3^ organized by MMTAs and MMPIs to reinforce students learning consisting of asking questions with multiple answers about the project. Finally, students and teachers and MMTAs received diplomas and awards for their contribution.

### Extramural Dissemination Activities

#### Science Fair *Expociencia*

A group of MMTAs and MMPIs attended every year the *Expociencia* science fair, promoted by the *Parc Científic* of the UV, held on the last Saturday of May. The aim was to show the project to all education levels using this platform. A multiple workshop consisting of two sections, an exhibition and another for handling samples, was designed. The exhibition workshop consisted of 5-min sessions, adapted to the age of the audience (children, teenagers, or adults) and showed the importance of the good use of antibiotics, as well as the present and future dangers of not doing so. Different explanatory panels adapted to the audience were prepared. In the second part, children and young people swabbed samples on TSA 1/10 plates. Participants were given a bookmark with a link where photos of the sown plates could be viewed later. Adults received an information card (business card format) to request additional information/visits to schools/research institutes if desired.

#### Activities in Favor of Gender Equality

A priority aim was to disseminate the project both at university and pre-university levels with a perspective of gender equality, taking advantage of the initiative #11 February (International Day of Women and Girls in Science). Under this scenario, university female students gave talks in high schools in the Valencian Community explaining the project, in terms of both its scientific basis and the importance of their own participation as scientists under training. After a presentation session, a debate with the speaker ran, asking questions about her experiences. The objective was 2-fold, to awaken scientific interest in the proposal and to motivate undergraduate students to choose science, technology, engineering, and mathematics (STEM) careers, since the analyses of the future labor market offer interesting perspectives of professional development for students of these degrees (Education and Training Monitor EU analysis, 2019).

#### Meeting on Teaching and Dissemination of Microbiology

Selected posters from the closing day and others, as well as oral communications based in the project or results obtained at the UV, were prepared by MMTAs under the supervision of their MMPIs, and were presented in the meeting of the D+DM group of the SEM in July 2018 at the UCM in Madrid (Spain).

#### Outreach Conferences

Conferences, round tables, and other activities were held in high/secondary schools located in the Valencian Community (in a radius of 150 km from UV) under the suggestion of the Scientific Culture and Innovation Unit Chair for Scientific Dissemination of UV. Lectures were followed by question/answer sessions in which students and teachers participated. In addition, training sessions were organized for secondary school teachers, also participating in monographic conferences in the Science Weeks running in towns of our Community or conference cycles in singular scientific centers (Institute of Biomedicine of Valencia, City of Arts and Sciences of Valencia, Planetarium of Castellón).

#### DivulSuperBugs

A group of students from the Biology degree at the UV prepared several infographics with information on the antibiotic crisis, antimicrobial resistance, and superbugs under the supervision of one of the participant professors. Infographics were submitted to a peer review system, where errors were reviewed and more information was provided when necessary. The following step was to establish teaching relationships among the infographics and create a cross-linked system to shape them as an exhibition. Two to three UV students acted as monitors of the exhibition in each school. Firstly, a brief explanation was given to the secondary school students and they could visit the infographics over a period of 1 week. Finally, direct learning was assessed using game-based classroom quizzes which engage students (Kahoot, escape rooms, etc.) designed by the monitors. Finally, anonymous surveys were run to evaluate the activity.

#### Media Coverage

The target audience of the previous activities was mainly adolescents. People in higher age ranges were accessible through other channels, such as conventional television, radio stations, or newspapers. In order to bring the problem of antimicrobial resistance closer to these age groups, specific collaborations were started with different public and private broadcasting companies.

**Figure 2:**
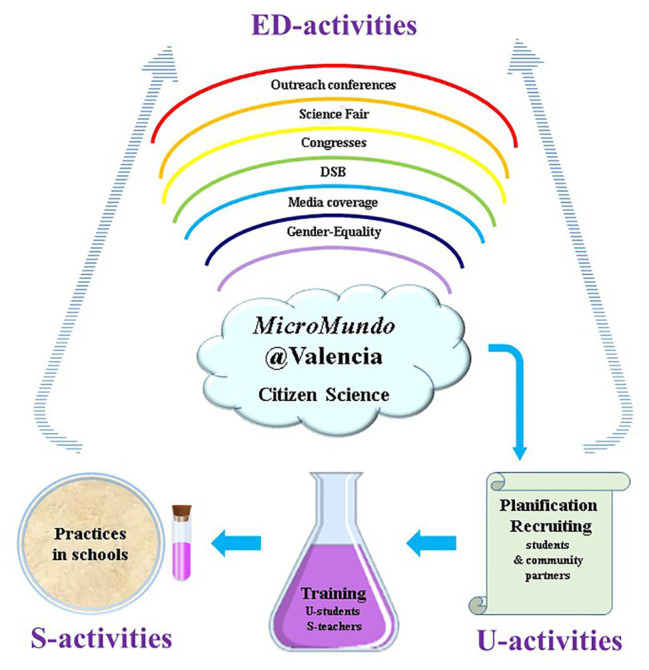
Flowchart of the different Service Learning and dissemination activities for the implementation of *MicroMundo*@Valencia in the Valencian Community. ED, extramural dissemination; S, school; U, university. Project activities in the Valencian Community began at the Universitat de València (U-activities) where university students (U-students) and school teachers (S-teachers) were recruited and trained to later adapt the activities to high/secondary and primary schools (S-activities). Project’s university and school activities were further disseminated in the Valencian Community through the implementation of diverse extramural activities (ED-activities) aimed at different age groups and with a gender perspective. Results, analyzed at the university, showed an increased sensitivity and awareness of the Valencian society to the global crisis of antibiotic resistance.

A summary of the different SL and dissemination activities carried out for the implementation of *MicroMundo*@Valencia in the Valencian Community is shown in Figure 2.

## Results and Discussion

Service Learning in microbiology courses is a teaching methodology that involves the participation of university and school students in research with the aim to improve their education and science interest as well as the community’s health (Webb, 2017). Two research-based programs focused on student discovery of novel soil microorganisms potentially producing new antibiotics, SWI, and TE, started in Spain in 2017 and 2018, respectively, under the current common name *MicroMundo*.

**Figure 3:**
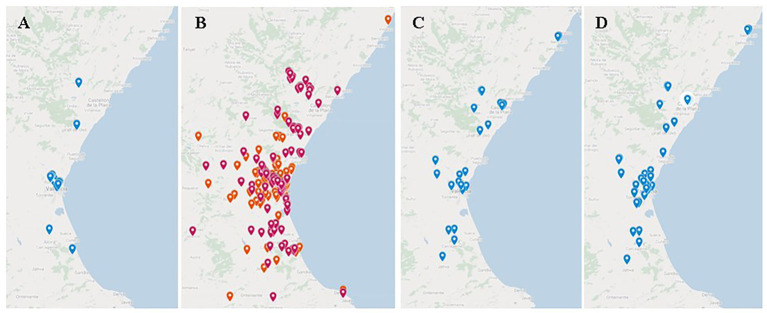
Location maps of some of *MicroMundo*@Valencia activities in the Valencian Community. **(A)** Discovery program in secondary schools (23). **(B)** Sampling points 2017/2018 (173) orange and 2018/2020 (193) pink. **(C)** Gender quality activities (30). **(D)** Conferences and weekly expositions (43).

The implementation of the *MicroMundo* project at the UV in the Valencian Community during the academic courses 2017–2018 and 2018–2019, allowed us to train up to 140 university students to disseminate the project to 23 high/secondary schools (Figure 3A) and one primary school in our influence area, involving some 900 people (teachers and students; Table 1). Particularly, in the course 2017–2018, 300 students and 14 teachers from 10 secondary schools from a network of public centers of the Valencian Community participated in *MicroMundo*@Valencia, as well as 66 MMTAs and 15 MMPIs from the UV. In the following academic course, 314 students and 17 teachers from 13 secondary schools were involved, together with 73 MMTAs and 10 MMPIs from the UV. The project also approached to elementary school in 2018–2019, involving 42 students (21 per classroom) and 2 teachers from a public primary school, 14 students and 1 teacher from a public high school, and 1 MMTA and 3 MMPIs from the UV.

**Table 1:** Professors and students involved in *MicroMundo*@Valencia initiative at the *Universitat de València* in the Valencian Community.

Academic course	MMPIs	MMTAs	Schools	Schools teachers	MMSs	Primary students
2017–2018	15	66	10	14	300	
2018–2019	10	73	13	17	314	
2018–2019 *Natura* project	3	1	1	3	14	42
Total	28	140	24	34	628	42

### Sample Collection and Isolation of Soil Microorganisms

**Figure 4:**
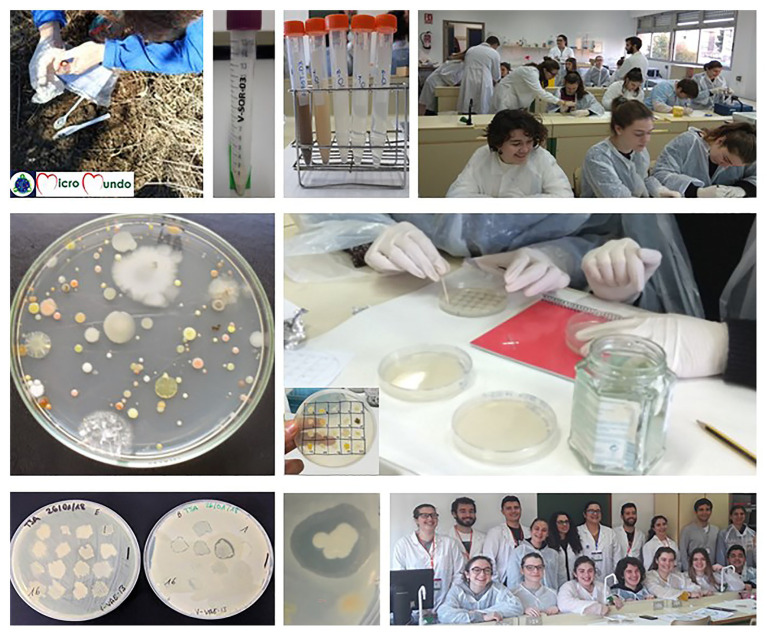
Implementation of *MicroMundo*@Valencia initiative at high/secondary schools in the Valencian Community. From left to right and from top to bottom representative photographs are shown about project activities, including soil sampling, diluting and plating of soil samples for isolation of microorganisms, observation of soil microbial diversity, and selection of colonies to test their antibiosis activities and results interpretation. Except for samplings that were carried out independently by the different groups of students over the weekend, the rest of activities were performed in the science laboratories of their respective centers. For further details, see “Materials and Methods” section.

The collection of soil samples and the isolation of microorganisms from these samples by the MMTAs and the students of the participating schools were performed in the first and the second semesters, respectively, of each academic year. In the case of MMTAs, samplings were done during the training course in November 2017 and November 2018, with three groups of 22–24 students each year. In the case of the school students, samplings were carried out along the months of February to April in 2018 and in 2019. During the first and second academic years, 173 and 193 soil samples were collected, respectively (Table 2). Samples were taken from soils of different geographical locations of the Valencian Community that were geolocated. The locations in 2017–2018 and 2018–2019 are presented in Figure 3B. Most soil samples were collected at a depth of between 0 and 2 cm (Figure 4), normally at temperatures ranging from 1 to 19°C in 2017–2018 and between 6 and 18°C in 2018–2019.

**Table 2:** Soil samples analyzed and results of *MicroMundo*@Valencia initiative at *Universitat de València* and high/secondary schools.

Course	Samples	Isolates	Antibiosis G−	Antibiosis G+
2017–2018	173	3,142	73	194
2018–2019	193	3,860	10	278
Total	366	7,002	83	472

Microbial counts were performed to estimate the heterotrophic bacteria in each one of the soil samples (Figure 4). Plate counts were carried out in a medium commonly recommended for the isolation of soil bacteria, diluted TSA containing only 1/10 of the nutrients (TSA 1/10; McCaig et al., 2001; Joseph et al., 2003), to favor the isolation of facultative oligotrophic bacteria from nutrient-poor environments such as soil. This medium was the same used previously by Valderrama et al. (2018) in SWI@Spain except that in our case it was not supplemented with the fungicide cycloheximide, since colony counts with bacterial morphology were performed after 72 h at room temperature. In our conditions, the plate counts of aerobic mesophilic heterotrophic bacteria obtained from the soil samples ranged from around 10^5^ to 10^7^ colony forming units per gram (CFU/g).

Since edaphic factors including pH, organic matter, texture, and carbonate contents, among others, influence microbial abundance (Mansour et al., 2015), some soil characteristics of a selection of samples were studied. The results revealed that most of the Mediterranean soils analyzed presented brownish coloration, sandy texture, and slightly alkaline pH values (7.2–8.5). Moreover, darker soils showed a higher content in organic material and, therefore, a higher content in microorganisms (Zhang et al., 2014). Soil samples with clay-like texture were those with higher number of CFU/g. These results agree with previous studies showing that cell numbers and microbial biomass are most concentrated in the smaller size silt and clay soil fractions (Sessitsch et al., 2001), which can be related with the influence of soil texture on nutrient retention and availability (Silver et al., 2000).

Soil isolates were tested by MMTAs or high/secondary students for their potential antibiosis activity against one standard Gram negative bacterium, *E. coli* CECT 101, and one standard Gram positive bacterium, *B. cereus* CECT 495. In the 2017–2018 course, from a total of 3,142 soil isolates tested, 73 (2.3%) showed growth inhibitory activity against *E. coli*, while 194 (6.2%) were active against *B. cereus* (Table 2; Figure 4). In 2018–2019, from 3,860 isolates tested, 10 of them (0.2%) were positive showing growth inhibition halos around the *E. coli* strain, whereas 278 (7.2%) showed inhibitory response against the strain of *B. cereus*. Taking into account the results of the antibiosis assays of the two courses together, from a total of 7,002 soil isolates tested, the percentages of isolates active against the *E. coli* or *B. cereus* strains were 1.2 or 6.4%, respectively. Only very few strains were active against both Gram-positive and Gram-negative bacteria. Although it is difficult to compare with the previous results of SWI@Spain by Valderrama et al. (2018) and de Groot et al. (2020) given that the number and type of soil isolates tested, as well as the control strains used for the antibiosis assays were different, the present results are similar in the sense that a higher percentage of soil strains with antimicrobial activity against Gram positive bacteria than Gram negative was obtained. In our case, the percentage of positive bacterial strains was higher than in the two previous studies which could be due, at least in part, to the higher number of soil isolates tested. This large number of soil isolates obtained as well as the relatively high percentage of them with potential activity against Gram positive or Gram negative bacteria has been made possible through the *MicroMundo*@Valencia initiative in which a large number of people have participated.

**Figure 5:**
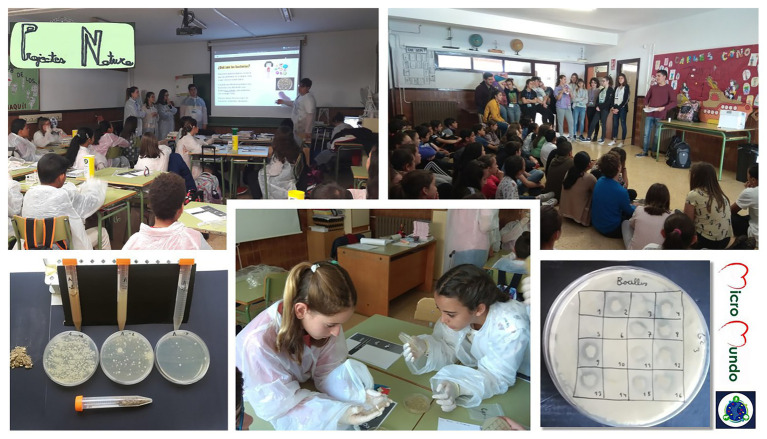
Implementation of *MicroMundo*@Valencia initiative at primary school within the framework of the *Natura* programme of the *Universitat de València*. In this innovative Service Learning program, teaching is given by university students to high school students and subsequently by the latter to primary school students, through adapted sessions, in their respective centers. Representative photographs of the activities of the project, including explanations and science practices (culturing soil bacteria and observations of microbial diversity and antibiosis). For further details, see “Materials and Methods” section.

Based on our results, the antibiosis tests at the primary school with a total of 320 soil isolates, after adapting the assay, were only carried out against the strain of *B. cereus* (Figure 5). In this case, 5.3% of soil isolates tested (17 out of 320) was positive showing an inhibitory halo of growth around the *B. cereus* strain. Thus, all our results confirmed that, similar to Valderrama et al. (2018) and de Groot et al. (2020), it was easier to find soil microorganisms able to inhibit Gram positive bacteria such as *B. cereus* than Gram negative bacteria such as *E. coli*. In this sense, it is expected that the soil bacteria tested show antibiosis activities against other soil bacteria such as *B. cereus* against which they may have to compete for space and/or resources to survive in microbial communities (Hibbing et al., 2010; Stubbendieck and Straight, 2016). Besides, it is more difficult to find soil bacteria producing antimicrobial substances against *E. coli*, whose natural habitat is the intestine. In addition, the outer membrane of Gram-negative bacteria is a semi-permeable barrier that protects them against different toxic compounds, including antimicrobials (Miller, 2016).

The antibiosis activity observed with some of the soil isolates was later confirmed in microbiology laboratories at UV. Those isolates that maintained their activity after purification were stored at −80°C for further studies since the two control bacteria used for the antibiosis tests were safe relatives to the bacterial pathogens grouped under the acronym “ESKAPE” (*Enterococcus faecium*, *Staphylococcus aureus*, *Klebsiella pneumoniae*, *Acinetobacter baumannii*, *Pseudomonas aeruginosa*, and *Enterobacter* spp.). ESKAPE bacteria are threatening nosocomial pathogens because of their resistance to most of the antimicrobial agents (Rice, 2008; Navidinia, 2016). In fact, they are in the World Health Organization (WHO) priority list of antibiotic-resistant bacteria against which new antimicrobials are needed (Tacconelli et al., 2018).

**Figure 6:**
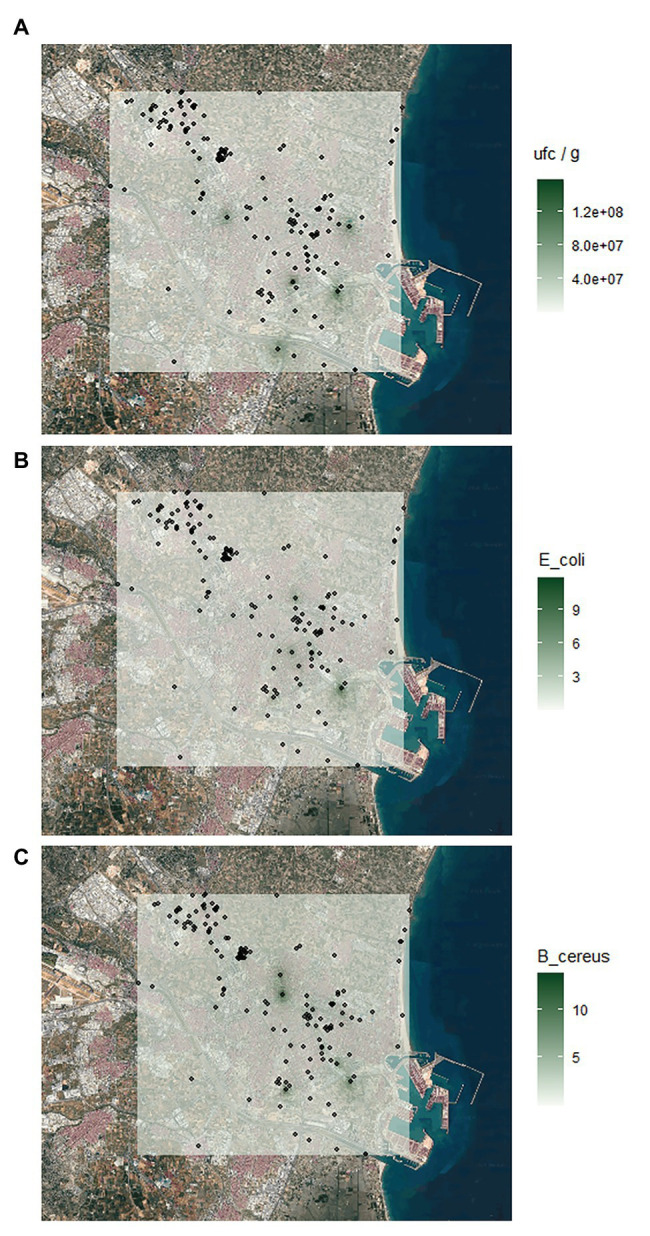
Kriging interpolation of the number of **(A)** colony forming units per gram (CFU/g) of soil, **(B)** soil isolates inhibiting *Escherichia coli*, and **(C)** soil isolates inhibiting *Bacillus cereus* in the metropolitan area of Valencia.

On the other hand, the geolocation of the soil samples allowed to draw a map of sampling sites (Figure 6), showing all the information available from the data collection form. With this information, different maps using Kriging technique were developed in order to show the spatial statistical presence of these bacteria. Figure 6A shows a map with the CFU/g as a colony density parameter of the elements that were available in each sample and interpolated to the metropolitan area of Valencia, not only the sampling points. This knowledge enables us to estimate in which areas we can find a higher level of bacteria and, later on, bacteria producing antibiotics. Further, the processed soil samples allowed gathering information of microorganisms able to inhibit the growth of control bacteria (*E. coli* or *B. cereus*). Thus, by performing a Kriging analysis, five soil foci that allowed the isolation of soil microorganisms able to inhibit the growth of *E. coli* and three foci of those able to inhibit the growth of *B. cereus* were detected (Figures 6B,C). These results suggest a potential application of geomapping to optimize antimicrobial research across large geographical regions to locate and predict the presence of antibiotic-producing microorganisms against *E. coli* and/or *B. cereus*.

Finally, the closing day of the *MicroMundo*@Valencia project was held in mid-May in each of the academic course in which it was carried out, to gather all the participating teachers and students. The objectives were to emphasize again the problem of antibiotic resistance by giving an informative talk, to show a summary of the results obtained in the project thanks to the effort made by all, and to promote the preparation and dissemination of results obtained through different activities and by conducting various competitions. As a result, a total of 13 videos, 25 posters, and 48 photos were presented to the assistants and numerous diplomas and prizes were awarded to the different participants.

### Evaluation of Activities at the University

**Figure 7:**
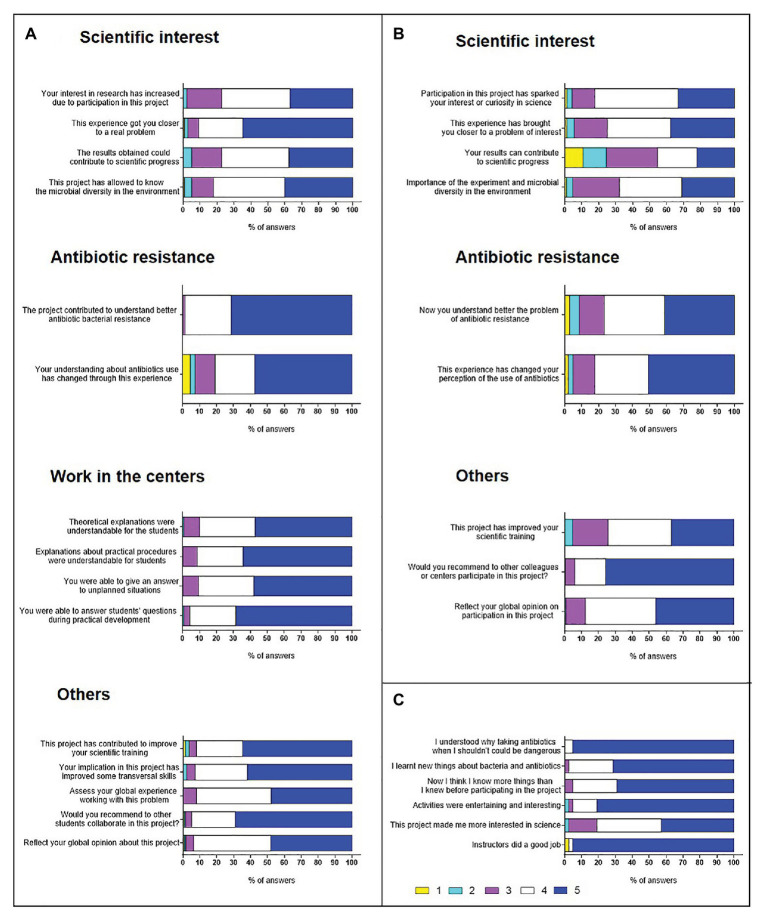
Two-year evaluation results of *MicroMundo*@Valencia activities by participating students in the Valencian Community. Anonymous surveys were carried out in the academic years 2017–2018 and 2018–2019, but the results are shown globally. The survey questionnaires were adapted according to the participation and educational level of the students. Response options were based on a 5-value Likert scale. **(A)** Surveys for *MicroMundo* Teaching Assistants (MMTAs; *n* = 140) consisted of 15 questions in four blocks in which they were asked about their opinion on the scientific interest of the project, their concept about antibiotic resistance, their experience as teachers for younger school students, and on their collaboration in the project. **(B)** Questionnaires for high/secondary school students (*n* = 628) included nine questions divided into three blocks to ask them about their scientific interest, their previous knowledge about antibiotic resistance, and their participation in the project. **(C)** Surveys for primary school students (*n* = 42) only included six questions about their learning experience, their interest in the project and their opinion on the high/secondary school students as monitors.

The *MicroMundo* Teaching Assistants’ perception of the *MicroMundo*@Valencia project based on the analysis of all surveys carried out in two academic courses was very positive, as most of the questions asked obtained the highest scores (4 and 5) on a 5-value Likert scale (Supplementary Material). The results, grouped into the four blocks of the questionnaire, are shown in Figure 7A. In relation to the scientific interest of the project, 77.8% of the students answered with 5 (37.1%) and 4 (40.7%), respectively, that their participation in the project increased their interest in science. Likewise, a 77.8% of them, with 5 (37.8%) and 4 (40%), respectively, also considered that their results could contribute to the scientific progress. It is noteworthy that around 90% and more than 80% of MMTAs (with scores of 4–5 points in both items), expressed that the project was targeting a real problem and allowed them to learn about microbial diversity in the environment, respectively. With regards to antibiotic resistance, all students considered that the project contributed to a better understanding of bacterial resistance to antibiotics, with 5 (71.4%) and 4 (27.1%) points, respectively. Most of them (80.8%), with 5 (57.3%) and 4 (23.5%) scores, also expressed that the experience improved their awareness of antibiotic use. Remarkably, survey results on MMTAs experience as teachers for high/secondary students in the schools were also very high, since from 57 to 68.8% of them gave 5 points in the four items. When rating 4–5 points together, 95.7% of the MMTAs knew how to answer the questions asked by the younger students during the science practices and 90.7% expressed that they were able to respond to unplanned situations. Similarly, 90 and 91.4% of university students (jointly recording scores 4 and 5) considered that practical and theoretical explanations, respectively, were carried out in a way that school students were able to understand. Regarding MMTAs’ opinion on their collaboration in the project in the remaining five questions of the block of “other questions,” it was very satisfying for more than 92% of them (rating 4–5 points in the five questions). In detail, three questions about whether the project had contributed to improve the MMTAs’ scientific training, their transferable competences, or if they would recommend participation in this project to other students, reached scores of 5 points in 64.7, 61.9, and 69%, respectively. Regarding the global assessment of students’ experience working on a real problem, the percentage of students that gave it a score of 5 to 4 was 47.8 and 44.2%, respectively. The last question in which the student had to reflect their global opinion on their participation in the project revealed very positive perception by MMTAs’ with 5 (48.1%) and 4 (45.7%) scores.

Other questions with free answer were made to the students that took part in the project in order to know their opinion about the best and worst things related to the activity and recommendations to improve. Regarding to the best things, MMTAs considered that it was a gratifying activity, since they learned new concepts and did a type of training different from the practices they did during their studies. Furthermore, they were pleased to transmit all these knowledge and experience to high/secondary students and to inform them and the rest of the community about the problem on antibiotic bacterial resistance. Another important point is that they were satisfied to see high/secondary students enjoying and learning doing a scientific practice, which could encourage them to follow scientific careers. Some MMTAs pointed out that it was a good experience to act as teachers for younger students. Conversely, among the worst things the MMTAs considered that some of the practical sessions at the high/secondary schools were overlapping with their classes at the university, so they had to lose some of them. Other things they complained were about the material shortage or that the sessions with high/secondary students were too short. Therefore, these MMTAs made some recommendations to improve project activities. They suggested shortening the length of the formation sessions and to use more and diverse materials, for example, different culture media and different bacterial strains.

### Evaluation of Activities in High/Secondary Schools

Survey results on students from high/secondary schools from two academic courses showed that they also liked the project activities. On a 1–5 scale, considering jointly the responses with scores of 4 and 5, results revealed that most students had a positive opinion in all three blocks of questions (Figure 7B and Supplementary Material). In the block on scientific interest, 82.3% of the students, with 5 (33.3%) and 4 (49%), considered that their participation in the project sparked their interest in science, 75%, with 5 (37.7%) and 4 (37.3%), that it brought them closer to a real problem and 67.8%, with 5 (31%) and 4 (36.8%), that the experience had an impact on their knowledge of microbial diversity in the environment. However, only 45.4% of the students expressed that their results can contribute to scientific progress (rating 4–5 points). Regarding to antibiotic resistance, 76.7% of the students, with 5 (41.2%) and 4 (35.5%), also expressed that their participation in the project contributed to a better understanding of the problem of antibiotic resistance and 82.3%, with 5 (50.6%) and 4 (31.7%), that this experience modified their perception of the use of antibiotics. In the third block of the questionnaire, considering together scores of 4 and 5, 74.2% of the students answered that the project improved their scientific training and 87.8% showed a very positive global opinion about their participation in the project. Remarkably, 94% of students, with 5 (75.8%) and 4 (18.2%), would recommend to other colleagues or centers the participation in this initiative. In the block of free response, more than 50% of the students commented that one aspect that could be improved would be to substitute the use of disposable plastic gowns, about 40% of them indicated that the sessions should have lasted a longer time, and about a quarter of them expressed that the worst part of the project was not obtaining positive antibiosis results. However, the results of the surveys reflected the didactic value of the sessions since the students highly valued the acquisition of the concepts related to them, as well as their participation in a scientific research process.

In addition to the social objective of this SL project, another one was learning and curricular complementation within the biology subjects, in which there is no specific microbiology block. However, the practical sessions are considered transferable skills, in that students put into practice and acquire several key competences. High/secondary school students developed mathematical competence, since part of the results obtained during the isolation of soil bacteria have to be expressed through mathematical calculations. In addition to the basic competences of the project, the participants acquired social and civic ones, making them aware of the public health problem posed by antibiotic resistance and, as a consequence, turning them into informed and critical citizens.

Moreover, in the context of the *Natura* project, secondary school students also developed digital competence since they had to use computer applications to transmit the project contents to primary students. As well as the competence of language communication to transmit the results through different channels using a specific language. Additionally, they carried out the competence of initiative and entrepreneurial spirit since they were the ones who chose the way of preparing and transmitting the information to students of lower educational levels.

Regarding surveys carried out by secondary school teachers to evaluate the impact of the project on their students, results were also very satisfactory. Teachers highly valued the complementation of the students’ practical skills. They also agreed on the suitability of these type of projects as a way to increase their students’ interest in science (data not shown).

Overall, survey results for university and high/secondary school students were very positive and similar to those of Valderrama et al. (2018) and de Groot et al. (2020).

### Evaluation of Activities in Primary Schools

Results of evaluation surveys filled by primary school students participating in the *Natura* project in the academic course 2018–2019 are shown in Figure 7C and in Supplementary Material. The evaluation of the youngest students of the project was also very positive based on their answers to the six questions of the survey, reaching 5 points with percentages of 69–95% in five of them, on the scale of 1–5. Based on the responses to the first three questions related to their learning through the project activities “I have understood when or not I have to take antibiotics,” “I have learned new things about bacteria and antibiotics,” and “I think I know more things than before participating in the project” with the highest score of 5 with percentages of 95.2, 71.4, and 69%, respectively, the elementary students gave a very high valuation to the project. Likewise, in the following two questions about students’ interest in the project activities and how this initiative has helped them become more interested in science, results revealed their high degree of satisfaction for 95.2 and 81% of them [rating 4 (14.2 and 38.1%) to 5 (81 and 42.9%) points in the two questions], respectively. Concerning the last question on whether the monitors did their job well, 95.2% of the primary school students rated it with 5 score.

Regarding the block of free response, about 85% of the students considered that they would not change anything in the activity, as well as that they really liked the theoretical and practical explanations. As negative aspects, a few students commented that they did not like the smell of bacterial cultures or having to wear a gown and gloves or considered that the sessions were too short. But, in general, primary students participating in the *Natura* project gave a very positive assessment of the scientific nature of the activity, as well as the acquisition of new knowledge and concepts related to the subject of natural sciences, which would reinforce the blocks of “Human beings and health” and that of “Living beings.” Additionally, in the practical sessions carried out with the primary students, they were able to develop several key competences, scientific ones, since they participated in the execution of the practices and, not less important, social and civic ones, by making them aware of a health problem that affects the society as a whole.

### Dissemination Activities

Different extramural dissemination actions were carried out over these 2 years within the *MicroMundo*@Valencia initiative. Firstly, around 500 children/teenagers, accompanied by their respective families or teachers, visited the *Expociencia* science fair and actively participated in the “antibiotics” workshop every year. They assayed an approach to the first step in the isolation of antibiosis producer strains whose results could be seen later by connecting to internet. In addition, hundreds of adults received theoretical explanations with the help of panels in order to raise their awareness on the rational and responsible use of antibiotics. Moreover, the adults were provided with an information card of the project, which encouraged several schools to contact us in order to join our initiative.

Secondly, we ran a novel extramural activity, an exhibition performed under the coverage of the specific subproject “DivulSuperBugs” (DSB). Thus, 14 infographics on the antibiotic resistance crisis, prepared by 42 students from the Biology degree at the UV, composed the exhibition which was coordinated by two or three university monitors in 14 secondary schools. The school students (average of 40 by center) visited it over a week and the monitors extended their involvement to a final session of direct learning using classroom quizzes. University students strongly engaged the younger students using the game-based learning platform Kahoot and escape rooms designed by themselves. In general, the school students highly valued the contribution of the exhibition to their understanding of bacterial resistance to antibiotics as well to become aware of their responsible use (data not shown).

Extramural scientific activities were performed under the perspective of gender equality. In fact, to strengthen this philosophy, university female students explained our project in around 30 high schools in the Valencian Community (Figure 3C). They highlighted their role as scientists under training and the promising professional perspectives for students of STEM degrees, trying to motivate secondary female students toward these university careers. In Europe, the demand for university degrees increases by 14% each year, however, only a very small percentage of students chooses to enroll in higher education linked to STEM (Rohaan et al., 2010; Education and Training Monitor EU analysis, 2019). The figures for women are even lower, anecdotal in some STEM degrees, due to a combination of social, cultural, and economic factors. That is why the general objective of the global STEM and Gender Advancement (SAGA) projects of UNESCO is to contribute to reduce the gender imbalance in STEM fields in all countries at all levels of education and research.^4^

As an important complement to inform a mainly young audience, we held over 40 conferences or round tables in regional high/secondary schools and contributed to the training of their science teachers (Figure 3D). We also participated in monographic conferences in Science Weeks running in towns of our Community (Gandia, Quart, …) and in singular scientific centers (Institute of Biomedicine of Valencia, City of Arts and Sciences of Valencia and Planetarium of Castellón).

In addition, within an academic context, several MMPIs and MMTAs attended the first SL *MicroMundo*@Valencia symposium held at the UCM in Madrid on July 2018, by presenting four oral communications and eight posters with the results of the project. Besides, our group also presented eight posters based on this SL experience on antibiotic resistance awareness in the IV Congress of the D+DM group of the SEM also held at the UCM. Two works presented by two MMPIs from the UV received awards, for the best poster and an *honorable mention*, respectively. Moreover, two MMTAs from BACPLANT group from UV, students of the master’s degree in Molecular, Cellular, and Genetics Biology, also received the award for the winning video of the competition “Do something about antibiotic challenge.” SWI/CDC/NIH/SEM (Link: https://youtu.be/q92S574-NoM). Throughout these years, we have collaborated with broadcasting companies (Supplementary Table S1), the most relevant contributions being two documentaries on the public television channel *À punt*,^5^ several radio programs,^6^ and a mini report in the newspaper with the largest circulation in our region.^7^ Moreover, numerous references have appeared in the local written press and radio stations. These diverse actions amply demonstrate the recognition achieved by the project in the academic context, as well as significant media coverage.

## Conclusion

The implementation of *MicroMundo*@Valencia initiative at the Universitat de València has increased the awareness of the public health problem posed by microbial antibiotic resistance in university, high/secondary and primary school teachers and students participating in the project in the Valencian Community, and has made possible their involvement in the active search for solutions. As a result, over 7,000 bacterial isolates were obtained from more than 300 soil samples, increasing the chances of finding new antimicrobials. In fact, some of these environmental isolates were able to inhibit the growth of target bacteria and could be tested against diverse pathogens in the future. In addition, geomapping of sampling sites combined with Kriging analysis allowed the localization of soil foci of antibiotic-producing bacteria, suggesting the potential of the application developed to optimize antimicrobial research across large geographical regions.

Evaluation of the project, based on student surveys, revealed that most of them acquired the expected scientific and pedagogical concepts and skills, improving their self-learning capacity and their academic and social engagement. This confirmed SL as a powerful teaching strategy that, in turn, allowed students involved to become a “transmission belt” to spread awareness of the problem of antibiotic abuse to society. Moreover, the project was able to promote student interest in science through the discovery of soil bacteria producing antimicrobials as well as to enable mutual cooperation among students of three different education levels. Further, the implementation of this initiative in a primary school, as a pilot experience, allowed the secondary and primary students involved to develop civic, social, and scientific competences, among others. Finally, *MicroMundo*@Valencia was also extended through additional outreach activities outside the university, and this generated sensitivity in the Valencian Community toward the global antibiotic crisis, while raising awareness of it.

## Data Availability Statement

The original contributions presented in the study are included in the article/Supplementary Material, and further inquiries can be directed to the corresponding author.

## Ethics Statement

Written informed consent was obtained from the individual(s) and minor(s)’ legal guardian/next of kin, for the publication of any potentially identifiable images or data included in this article.

## Author Contributions

All authors participated in the implementation of *MicroMundo*@Valencia at the UV and in high/secondary schools, and SM, ÀF-S, and EB also in a primary school. JS-G developed the APP for geolocate sampling sites. EC determined properties of soil samples. SM, BF, ÀF-S, HR, and EB participated in extramural activities. SM, BF, and EB conceived and designed the manuscript. All authors contributed to the manuscript by drafting the work or revising it. SM, BF, ÀF-S, HR, JS-G, and EB generated figures and/or tables. SM, ÀF-S, EC, JS-G, and EB analyzed the data. All authors approved the final version of the manuscript.

### Conflict of Interest

The authors declare that the research was conducted in the absence of any commercial or financial relationships that could be construed as a potential conflict of interest.

## Footnotes

^1^http://swispain.blogspot.com/2017/07/i-workshop-swispain-2017-el-programa.html

^2^https://www.uv.es/swi/manualapk.pdf

^3^http://kahoot.com

^4^http://www.unesco.org/new/en/natural-sciences/priority-areas/gender-and-science/improving-measurement-of-gender-equality-in-stem/

^5^https://apuntmedia.es/va/noticies/punt-docs/21-12-2018-l-amenaca-dels-bastonets and https://vimeo.com/388287770

^6^https://vimeo.com/388287770 and https://www.ivoox.com/a-ciencia-cierta-18-11-2019-la-verdad-sobre-los-audios-mp3\_rf\_44430590\_1.html

^7^https://www.levante-emv.com/aula/2018/02/21/joves-recerca-nous-antibiotics/1681964.html
