# Supplementary material for: Implementation of Antibiotic Discovery by Student Crowdsourcing in the Valencian Community Through a Service Learning Strategy
Source: Front Microbiol. 2020 Nov 16;11:564030. doi: 10.3389/fmicb.2020.564030 (PMC7702300; doi:10.3389/fmicb.2020.564030)
Supplement: Supplementary file 2 [file Table_1.docx]

**Table S1. Media coverage in public and private broadcasting systems.**

| **Activity** | **Publisher** | **Date** | **Link** |
| --- | --- | --- | --- |
| Interview | Ràdio Klara | 2018 | <http://audio.urcm.net/La-Luna-Sale-a-Tiempo-Radio-09-02> |
| Documentary | À punt (Television) | 2018 | <https://apuntmedia.es/va/a-la-carta/programes/vist-en-tv/punt-docs/21-12-2018-l-amenaca-dels-bastonets> |
| Interview | Ràdio Klara | 2018 | <http://audio.urcm.net/La-Luna-Sale-a-Tiempo-Radio-26-01> |
| Documentary | Levante-EMV (Journal) | 2018 | <https://www.levante-emv.com/aula/2018/11/14/formula-dels-nous-antibiotics-lestudiantat/1794708.html> |
| Interview | Aulacodi (Electronic Journal) | 2018 | <https://www.aulacodi.cat/noticia/52/small-world-initiative-un-projecte-de-ciencia-ciutadana-per-fer-front-a-la-ineficacia-dels> |
| Interview | La Cotorra de la Vall (Electronic Journal) | 2018 | <http://lacotorradelavall.blogspot.com/2018/05/lies-jaume-ii-el-just-ha-participat-en.html> |
| Documentary | Levante-EMV (Journal) | 2018 | <https://www.levante-emv.com/aula/2018/02/21/joves-recerca-nous-antibiotics/1681964.html> |
| Interview | La Cotorra de la Vall (Electronic Journal) | 2019 | <http://lacotorradelavall.blogspot.com/2019/01/lies-jaume-ii-el-just-va-acollir-una.html?m=1> |
| Documentary | À punt (Radio) | 2020 | <https://vimeo.com/387999891> |
| Documentary | À punt (Television) | 2020 | <https://vimeo.com/388287770> |
| Interview | La Llosa Radio FM | 2020 | <https://www.ivoox.com/micro-obert-programa-38-xerrada-sobre-audios-mp3_rf_47262830_1.html> |
| Interview | El Temps (Journal) | 2020 | <https://www.eltemps.cat/article/9851/superbacteris-o-com-la-resistencia-als-antibiotics-pot-causar-pandemies> |
